# Source Apportionment and Model Applicability of Heavy Metal Pollution in Farmland Soil Based on Three Receptor Models

**DOI:** 10.3390/toxics11030265

**Published:** 2023-03-13

**Authors:** Jiawei Ma, Kaining Lanwang, Shiyan Liao, Bin Zhong, Zhenhua Chen, Zhengqian Ye, Dan Liu

**Affiliations:** 1 Key Laboratory of Soil Contamination Bioremediation of Zhejiang Province, Zhejiang A & F University, Lin’an 311300, China; 2 Department of Applied Engineering, Gandong University, Fuzhou 344000, China; 3 Hangzhou Zhonglan Shunong Ecological Technology Co., Ltd., Lin’an 311300, China; 4 Jingning Agricultural and Rural Bureau, Lishui 323000, China

**Keywords:** heavy metals, source apportionment, APCS-MLR, UNMIX, PMF

## Abstract

The identification of the source of heavy metal pollution and its quantification are the prerequisite of soil pollution control. The APCS-MLR, UNMIX and PMF models were employed to apportion pollution sources of Cu, Zn, Pb, Cd, Cr and Ni of the farmland soil in the vicinity of an abandoned iron and steel plant. The sources, contribution rates and applicability of the models were evaluated. The potential ecological risk index revealed greatest ecological risk from Cd. The results of source apportionment illustrated that the APCS-MLR and UNMIX models could verify each other for accurate allocation of pollution sources. The industrial sources were the main sources of pollution (32.41~38.42%), followed by agricultural sources (29.35~31.65%) and traffic emission sources (21.03~21.51%); and the smallest proportion was from natural sources of pollution (11.2~14.42%). The PMF model was easily affected by outliers and its fitting degree was not ideal, leading to be unable to get more accurate results of source analysis. The combination of multiple models could effectively improve the accuracy of pollution source analysis of soil heavy metals. These results provide some scientific basis for further remediation of heavy metal pollution in farmland soil.

## 1. Introduction

Soil is an important material basis for agricultural production and human survival [1]. In recent decades, with the gradual increase in population and rapid development of industry and agriculture, the problem of heavy metal pollution in the farmland soil of China has become a crucial problem [2,3]. Heavy metals in farmland soil are highly related to the safety of agricultural products, which has attracted worldwide public attention [4,5]. Heavy metals can not only threaten soil quality and ecological environment but also be accumulated in human body with transmission through the food chain [6,7]. Measures should be taken to control and remediate heavy metal pollution in farmland soil, which is essential to ensure the quality of the soil environment and the safety of agricultural products. Furthermore, assessment of the status and sources of soil heavy metal pollution is a prerequisite for effective prevention of pollution.

Heavy metals in soil mainly originate from soil parent materials and human activities. Heavy metals contained in parent materials are enriched in the soil by weathering and leaching [8]. Human activities mainly include high-intensity industrial and mining activities, agricultural activities and transportation that contribute heavy metals to farmland soil through atmospheric deposition, sewage irrigation, fertilizer input and solid waste [9,10,11]. Existing methods of the source apportionment of heavy metals can be roughly divided in two categories: one is qualitative source identification, and the other is quantitative source apportionment [12]. The former mainly uses multivariate statistical analysis (principal component analysis, cluster analysis, correlation analysis, etc.) and geostatistical analysis to identify major pollution sources [13,14,15]. The latter uses physical and chemical characteristics of pollutants in receptors to identify pollution sources and to quantify its contribution rate [12]. Among receptor models, absolute principal component scores–multivariate linear regression (APCS-MLR), UNMIX and positive matrix factorization (PMF) were widely used in the source apportionment of heavy metals in soils [16,17,18]. The model APCS-MLR combines factor analysis and multiple linear regression to quantify pollution sources, with simple operation and fast calculation speed [19]. The UNMIX model automatically removes unreasonable data through the system built in the model and does not need to set the number and uncertainty of pollution sources, which reduces the impact of human factors [20]. The PMF model limits the factor scores and factor loads to be non-negative during the solution process, which can handle missing and inaccurate data [21]. The receptor models are widely used to perform source apportionment of atmosphere, water, sediment and soil [20,22,23,24].

The research on the source apportionment of heavy metals has mainly focused on the analysis of potential pollution sources and their contributions in contaminated sites; however, the applicability of receptor models is rarely considered. Since soil is not a homogeneous matrix, it has a high degree of spatial heterogeneity [25]. The types, quantity and pollution status of pollution sources are different in different regions. The analytical results of a receptor model are affected by factors such as sample data value errors and modeling errors; using a single receptor model that cannot obtain information of pollution sources accurately makes its conclusions controversial. Therefore, this study has employed several commonly used source analysis methods to analyze the sources of heavy metals and to assess the potential risks of heavy metals in farmland soils. The variation between validation models was compared and its causes were analyzed to accomplish more reliable results for source apportionment and to set a perfect foundation for prevention and control of heavy metal pollution in farmland soil.

The objectives of this study were (1) to investigate the soil heavy metal pollution in the study area, (2) to analyze the sources and contributions of heavy metals in the study area by using the APCS-MLR, UNMIX and PMF models and compare the applicability of the models, and (3) to evaluate the potential impact of heavy metal pollution in the study area in terms of ecological risk.

## 2. Materials and Methods

### 2.1. Study Area

**Figure 1:**
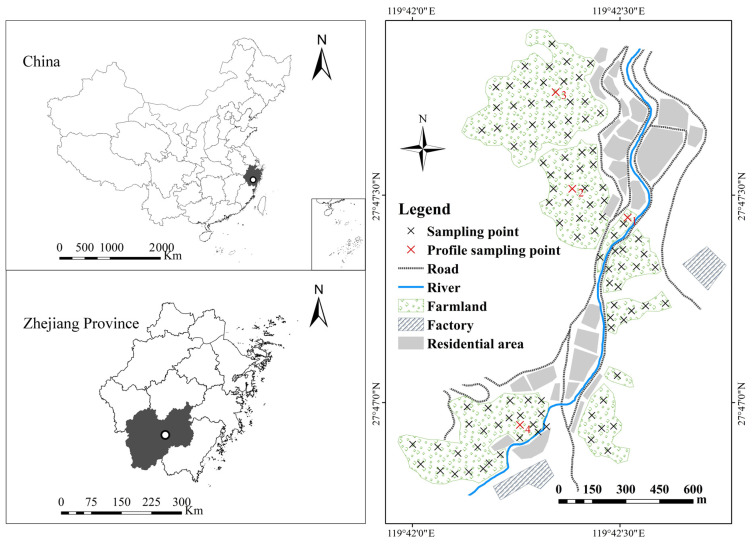
Distribution of sampling sites in the study area.

The study area is located in the southeast of China, the town of Lishui City, Zhejiang Province. The study area covers an area of 2.12 km^2^, ranging from 119°42′44.7′′ to 119°42′5.5′′ E and 27°46′50.6″ to 27°47′54.3″ N. The study area belongs to a subtropical monsoon climate zone, with an annual average temperature and precipitation of 17.8 °C and 1568.4 mm, respectively. A northeast wind prevails in the study area. The farmland in the study area is in the vicinity of the township residential area. A waste steel plant is located in the south of the study area, and there is a bamboo processing plant in the east (Figure 1). The farmland is terraced field, and a river flows from south to north. The water for farmland irrigation mainly comes from rainfall and river water pumping irrigation. The main crop in the study area is single cropping rice, and according to WRB 2022 the, soil taxonomy in the studied area is hydragric.

### 2.2. Sample Collection and Preparation

According to a grid density of 50 m × 50 m, 101 surface soil samples (0–20 cm) were collected. Each soil sample was composed of 5 subsamples, which consisted of one from the center and four from the surrounding area. The samples were put into PVC bags and the coordinates of the sampling points were recorded. The collection of four profile samples was consistent with the collection of soil surface samples, which consisted of five sub-samples, one from the center and four surrounding ones 5 m apart. The sampling point map is shown in Figure 1. The soil samples were air-dried in natural conditions and visible intrusion of plants in samples were removed. The air-dried soil samples were passed through a 2 mm aperture sieve. The grounded samples were passed through a 100 mesh sieve and stored in sample bags. The soil pH was measured by pH meter (FE28, Mettler Toledo, Shanghai, China) with a soil water ratio of 1:2.5. Soil organic matter (SOM) was determined by heating a mixture of potassium dichromate and concentrated sulfuric acid at 180 °C, and then titrated with ferrous sulfate solution. The content of soil heavy metals was determined by the mixtures of HF (7 mL)—HNO_3_ (5 mL)—HClO_3_ (1 mL) to a fixed the volume of 50 mL with de-ionized water. The digested solution was washed in 50 ml flasks and volume was made using de-ionized water. The contents of copper (Cu), zinc (Zn), lead (Pb), chromium (Cr) and nickel (Ni) were determined by inductively coupled plasma optical emission spectroscopy (ICP-OES, Leeman prodigy 7 Hudson, NH, USA). The cadmium (Cd) was analyzed by graphite furnace atomic absorption spectrometry (GFAAS, PerkinElmer AA800, Los Angeles, CA, USA). The number of the certified reference material was GBW 07405 (GSS-5) for the verification of soil analysis.

### 2.3. Source Apportionment Models

#### 2.3.1. Absolute Principal Component Scores–Multivariate Linear Regression (APCS-MLR)

The APCS-MLR model was proposed by Thurston and Spengler in 1985. According to results of principal component analysis, the factor score was transformed to normalized factor score, and multiple linear regression was performed on receptor content. The contribution rate of the pollution source corresponding to each factor of the substance in the receptor was calculated with the regression coefficient as follows.

(1) $$Z_{ij}=\frac{C_{ij}-\bar{C_{i}}}{\sigma_{i}}$$

(2) $${\left(Z_{0}\right)}_{i}=\frac{0-\bar{C_{i}}}{\sigma_{i}}=-\frac{\bar{C_{i}}}{\sigma_{i}}$$

(3) $$APCS_{p}={\left(Z_{0}\right)}_{i}-Z_{ij}$$

(4) $$C_{i}=b_{0i}+\overset{n}{\underset{p=1}{\sum }}\left(APCS_{p}\times b_{pi}\right)$$

where *C_ij_* is the content of heavy metal *i* at the *j*th sampling point; $\bar{C_{i}}$ is the mean content;$\;\sigma_{i}$ is the standard deviation; $Z_{ij}$ is normalization matrix of element content; ${\left(Z_{0}\right)}_{i}$ is the factor score of the “zero” pollution point where all element contents are equal to 0. The principal component analysis was conducted for $Z_{ij}$ and ${\left(Z_{0}\right)}_{i}$; *p* is the number of factors obtained in principal component analysis. $APCS_{p}$ is the absolute principal component scores;$\;b_{0i}$ is a constant term obtained by multiple linear regression for metal element *i*;$\;b_{pi}\;$ is the linear regression coefficient of element *i* on factor *p*. The contribution of each source was calculated by $b_{pi}$ and $\;APCS_{p}$.

#### 2.3.2. UNMIX Model

The UNMIX model is a receptor model developed by the U.S. Environmental Protection Agency. Based on the contribution of different pollution sources to the receptor (soil), it is a linear combination of different source components [26]. The equation is listed below:

(5) $$C_{ij}=\overset{m}{\underset{k=1}{\sum }}F_{jk}S_{jk}+E$$

where the *C_ij_* is the content of heavy metal *i* in the *j*th sampling point, *F_jk_* is the percentage of element *j* in the *k* source, *S_ik_* is the contribution of *k* source in sample *i* and *E* is the standard deviation of analysis. The source component spectrum parsed by the model needs to meet minimum system requirements that can be interpreted by the model (Min Rsq > 0.8, Min Sig/Noise > 2).

The UNMIX model was standardized before importing data to the model. The data were dimensionless and value range of observation was between 0 and 1. The standard formula of dispersion was as follows [27]:

(6) $$X_{k}=\frac{X_{i}-X_{i\;min}}{X_{i\;max}-X_{i\;min}}$$

where *X_k_* (*k* = 1, m) is the value after deviation standardization, *X_i_* is the initial analysis value of the sample, *X_i min_* is minimum analysis value and *X_i max_* is maximum analysis value.

#### 2.3.3. Positive Matrix Factorization (PMF)

The PMF model is a multivariate factor analysis tool that decomposes the receptor concentration data matrix into a factor contribution matrix and a factor distribution matrix under non-negative constraint [28]. The goal of PMF is to analyze pollution sources and source contributions based on synthetic data sets [29]. The calculation method is as follows:

(7) $$X_{ij}=\overset{p}{\underset{k=1}{\sum }}G_{ik}F_{kj}+E_{ij}$$

where *X_ij_* is concentration matrix of the *j*th heavy metal in the *i*th sample, *G_ik_* is the contribution of the *k*th source to sample *i*, *F_kj_* is the value of the *k*th source to concentration of heavy metal *j* and *p* is the number of factors; *E_ij_* is the residual.

The PMF model is defined and iterated on the basis of a weighted least squares method. The factor contribution and distribution were obtained by minimizing the objective function Q of the PMF model [30]. The calculation method is as follows:

(8) $$Q=\overset{n}{\underset{i=1}{\sum }}\overset{m}{\underset{j=1}{\sum }}{\left(\frac{E_{ij}}{U_{ij}}\right)}^{2}$$

where *U_ij_* is uncertainty of element *j* in sample *i*.

The PMF model was run with concentration data and uncertainty data. The uncertainty data includes sampling and analysis errors [31]. In this study, the contents of Cu, Zn, Pb, Cd, Cr and Ni in the soil were all higher than the detection limit *MDL*. The calculation method of uncertainty *Unc* was as follows:

(9) $$U_{nc}=\sqrt{{\left(\theta \times C_{ij}\right)}^{2}+{\left(MDL\right)}^{2}}$$

where *C_ij_* is concentration of heavy metal *i* in the *j*th sample; *MDL* is the detection limit of the sample; *θ* is the relative standard deviation; the signal to noise ratio (S/N) can be calculated by PMF, S/N > 2 can be considered as good data quality, and samples with 0.2 < S/N < 2 can be considered as poor data quality, unable to provide sufficient concentration change [32].

### 2.4. Potential Ecological Risk Index

The potential ecological risk index (*RI*) combines ecological and environmental effects of heavy metals with toxicology [33]. The calculation formula for *RI* is as follows:

(10) $$E_{r}^{i}=T_{r}^{i}\times \left(\frac{C_{i}}{S_{i}}\right)$$

(11) $$RI=\overset{n}{\underset{i}{\sum }}T_{r}^{i}\times \left(\frac{C_{i}}{S_{i}}\right)$$

where $T_{r\;}^{i}$ is the biological toxicity of different elements *i*, which was determined as Zn = 1 < Cr = 2 < Cu = Ni = Pb = 5 < Cd = 30. The *C_i_* is the measured value of heavy metal *i* (mg·kg^−1^), *S_i_* is the reference value of heavy metal *i* in soil (background value in Zhejiang Province). $E_{r}^{i}$ represents the potential ecological risk factors. *RI* is the comprehensive potential ecological risk index of heavy metals. $E_{r}^{i}$ < 40, *RI* < 150, indicates low potential ecological risk; 40 ≤ $E_{r}^{i}$ < 80, 150 < *RI* < 300 indicate moderate potential ecological risk; 80 ≤ $E_{r}^{i}$ < 160, 300 ≤ *RI* < 600 shows considerable potential ecological risk; 160 ≤ $E_{r}^{i}\;$ < 320, 600 ≤ RI < 1200 exhibit high potential ecological risk; and $E_{r}^{i}$ ≥ 320, *RI* ≥ 1200 reveal very high potential ecological risk.

### 2.5. Data Processing and Statistical Analysis

The soil sampling points in the study area were located with ArcGIS 10.2. The descriptive analysis, correlation analysis and APCS-MLR model calculation was conducted with SPSS 22.0. The analysis chart was drawn with Origin version 8.5. The source apportionment of heavy metals was determined with the PMF model (EPA PMF 5.0) and the UNMIX model (EPA UNMIX 6.0).

## 3. Results and Discussion

### 3.1. Descriptive Statistical Analysis of Heavy Metals in Soil

Table 1 reveals the descriptive statistical analysis of heavy metals (Cu, Zn, Pb, Cd, Cr and Ni) and physical–chemical properties of farmland soil in the study area. Soil was acidic, as its pH ranged from 4.0 to 5.4. The mean contents of Cu, Zn, Pb, Cd, Cr and Ni were 30.3, 271.5, 151.4, 0.4, 67.8 and 29.1 mg·kg^−1^, respectively. The average contents of heavy metals in the study area were 1.7, 3.9, 6.4, 5.3, 1.3 and 1.2 times their background values, respectively, compared with the background values of Zhejiang Province [34]. The over standard rates of Cu, Zn, Pb, Cd, Cr and Ni in soil were 3.96%, 86.14%, 97.03%, 70.30%, 0.00% and 0.99%, respectively, compared with the screening values of agricultural soil environmental risks in China (GB 15618-2018). The coefficient of variation (CV) reflects the variability and dispersion of heavy metal elements in soil. The strong variability indicates that the spatial distribution of heavy metals was seriously affected by external factors [35,36]. The CVs of heavy metals can be classified as follows: CV ≤ 15% means weak variation; 15% < CV < 36% means moderate variation; and CV ≥ 36% means high variation. The CV of Pb, Cd and Ni in the study area were 38.5%, 40.17% and 39.03%, indicating high variation. The elements of other heavy metals showed moderate variation. This shows that external factors have a significant impact on the accumulation of heavy metals in soil.

### 3.2. Heavy Metal Content in Soil Profile

**Figure 2:**
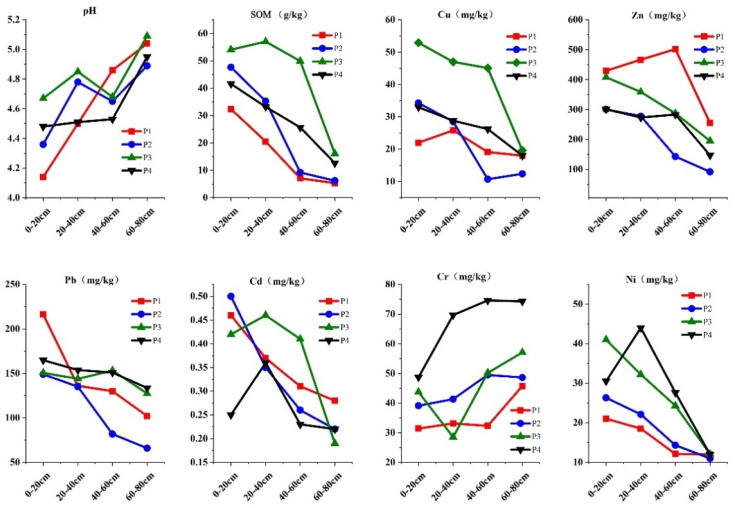
Distribution of heavy metals, pH and organic matter in soil profiles.

For the collected soil profile samples, the diagnostic horizons of the four sections were divided into tillage layer (A), plough bottom layer (P), percolation layer (W) and sediment layer (B). In the upper layer, there were many roots with high porosity and the soil was dark brown. In the lower layer, there was no root system. The soil layer was compact with rust patterns and spots and a small amount of iron and manganese nodules. We further analyzed the soil texture at different depth levels. There are significant differences among the soil profiles (Table 2), likely affecting the migration of heavy metal elements. Figure 2 demonstrates the depth distribution of soil pH, organic matter (OM) and heavy metals in the soil profiles. The pH value of surface (0–20 cm) soil was lowest compared with highest of bottom (60–80 cm) soil. Excessive use of nitrogen fertilizer has led to serious acidification of agricultural soil in China. Nitrogen fertilizer has been identified as the main driving force of acidification in farmland soil by affecting the process of nitrogen transformation [37,38]. The content of SOM decreased with the increase in depth. This was mainly attributed to the application of fertilizer and animal manure in farmland soil, being mainly accumulated in the surface layer and then transferred from the surface layer to the deeper soil after decomposition [39,40]. The contents of Cu, Zn, Pb, Cd and Ni in surface soil were significantly higher than in deep soil (40–60 cm) and bottom soil (60–80 cm) soil, which indicated that these heavy metals were greatly affected by the input of external activities. The content of Cr in the bottom (60–80 cm) soil was significantly higher than surface soil (0–20 cm), which indicated that Cr was mainly affected by soil parent material. The content of SOM and Cu in P3 were enriched at 0–60 cm, which were significantly higher than the other three profiles. Animal manure and its processed products (organic fertilizer) were the important reasons for the significant increase in the content of organic matter and Cu in soil, indicating that the region was greatly affected by agricultural activities [41]. However, the Zn and Pb contents in P1 were significantly higher than in the other three sections, which has confirmed the sources of potential heavy metal pollution in this area.

### 3.3. Results of Three Models

The source distribution of heavy metals in the study area was analyzed with the APCS-MLR, UNMIX and PMF models. The APCS-MLR had extracted four factors, accounting for 88.58% of the data variance. The explained variances were 25.13%, 25.0%, 20.91% and 17.6%. The PMF model had set the number of factors as four to seven and the number of runs was 20. The optimal number of factors was finally determined to be four after comprehensive trial calculation of the Q value. The UNMIX model resolved four factors, among which both Min Rsq = 0.95 and Min Sig/Noise = 2.03 met the minimum value required by the system (Min Rsq > 0.8, Min Sig/Noise > 2).

**Figure 3:**
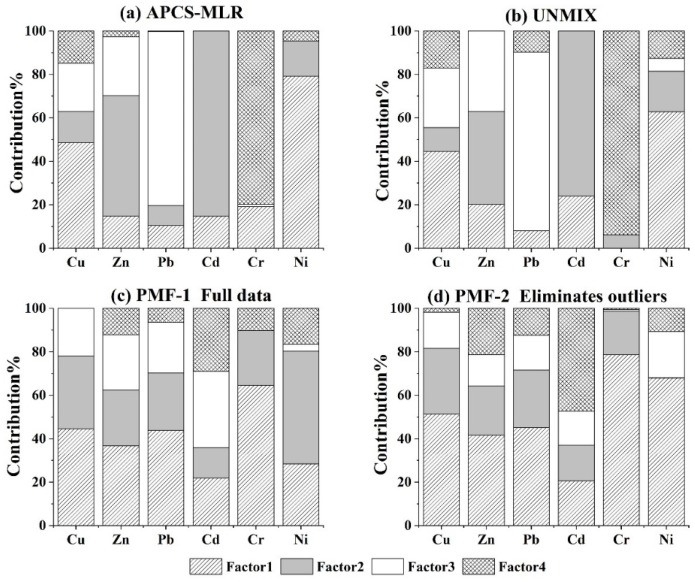
Source factor analysis of soil heavy metals in the study area.

Figure 3a–c exhibits the composition of factors as analyzed by the APCS-MLR, PMF and UNMIX models. The factor components of the APCS-MLR and UNMIX models were similar. In factor 1, Cu and Ni had higher loads. Factor 2 was Cd and Zn, factor 3 was Pb and factor 4 was Cr. In addition, the fitted parameters with three models were described in Table 2. The minimum value of r^2^ was Cu (0.79) in APCS-MLR, and the r^2^ of the other heavy metal elements was greater than 0.8 both in APCS-MLR and UNMIX, revealing the ideal fitness of the two models. In the PMF model, the factor composition was quite different from APCS-MLR and UNMIX. Factor 1 was dominated by Cu, Zn, Pb, Cd and Ni; factor 2 was Cu, Zn, Pb, Cr and Ni; factor 3 was Cd; and factor 4 was Cr. The r^2^ between the predicted and observed values of Cu, Cd and Ni in PMF was close to 1 according to the component fitting results (Table 3). The r^2^ of Zn, Pb and Cr were 0.402, 0.119 and 0.403, respectively, whereas the fitness of the results was not ideal. The studies revealed that the PMF model was abnormally sensitive to outliers and cannot acquire reasonable results without the elimination of outliers.

The 13 sampling points were deleted according to the unified residual value of each point until *Q_robust_* and *Q_ture_* were in proximity to unreliable results of source analysis using the complete data in PMF. Four factors were obtained in PMF, and the factor composition is presented in Figure 3d. The proportion of factor 1 in each heavy metal element increases compared with complete data, whereas the proportion of factor 3 decreases after excluding the abnormal values. The fitness of the model has improved the r^2^ values of Zn, Pb and Cr and the observed r^2^ values were 0.448, 0.213 and 0.61, respectively. However, no more reliable fitting results were obtained, which is consistent with results of Xue et al. [30].

### 3.4. Source Apportionment of Heavy Metals in Soil

The composition of pollution sources in the APCS-MLR and UNMIX models was similar. In the composition spectrum of factor 1 (Figure 3), the load of Cu and Ni was higher than other elements. The Zn, Pb, Cd and Cr were distributed in a small amount. Research has reported that human activities could significantly change the spatial characteristics of Ni in soil and diffusion of Ni in the environment was mainly affected by atmospheric deposition and sewage irrigation [42,43]. In industrial enterprises, the iron and steel plant was a significant source of heavy metal pollution. In the past few decades, due to lack of environmental protection technology to address the dust and waste produced in the iron and steel smelting process, the Cu content in the surrounding soil was five times higher than the background value [44]. The study of heavy metal content in soil near a steel plant in Serbia showed that concentrations of Cd, Cu, Ni, Pb and Zn were higher than that reported in European soils and higher than the world average [45]. The abandoned iron and steel plant was located in the north of the study area, in the upper reaches of the river, and the terrain was higher than the farmland. Dust and sewage generated in the process of steel smelting and processing were accumulated in soils with atmospheric sedimentation, rain erosion and farmland irrigation. Therefore, factor 1 represented the industrial pollution source.

In factor 2, the calculation results of the APCS-MLR and UNMIX models suggested that Cd and Zn were marker elements (Figure 3a,b). Pearson correlation analysis results among heavy metal content and physical–chemical properties in soils are reported in Table 4. SOM had a significant correlation with Cd and Zn at *p* < 0.05, and its correlation coefficients were 0.487 and 0.437, respectively. As discussed in the previous sections, Cd usually exists in phosphate rock and as chemical fertilizers, especially phosphate fertilizers, brought to farmland soil [46]. Zn is widely used in livestock feed as additives, which is excreted out of the body with feces [47]. Long-term application of chemical fertilizer, organic fertilizer and animal manure in agricultural activities will enhance the concentrations of Zn and Cd in soil [48,49]. According to the field survey, the farmers bred livestock and poultry directly on the farmland after harvest, which transferred heavy metals to soil from the manure of the livestock. In addition, this was an important reason why the content of SOM in this study was significantly higher than the average level of Zhejiang Province. In this regard, factor 2 could be identified as an agricultural source.

Factor 3 represents transportation, which is major cause of pollution due to Pb, Cu and Zn. Due to the combustion of fuels and use of engine catalysts, Pb was main indicator of traffic emissions [50,51]. Although the production, sale and use of leaded gasoline has been banned since 2000, the accumulation of Pb in soil has not been eliminated [52]. Cu is usually used in the metal parts of automobiles. The Zn in surface soils is related to the wearing of tires and corrosion of galvanized parts [53,54]. In this study area, a large number of farms are distributed along the road. The major cause of Pb, Cu and Zn pollution in farmland soil was due to road dust. Furthermore, in profile 1 (Figure 2), the Pb content in topsoil was significantly higher than in the other three sections of soil. The heavy metal (Pb) in the soil was enriched by intensive transportation. In factor 4, the ratios of Cr determined by the APCS-MLR and UNMIX models accounted for 79.75% and 93.37% of the totals, respectively. The concentration of Cr in the study area was significantly lower than the background value. According to the soil profile analysis (Figure 2), Cr in the soil originated from parent material, which has been verified in previous studies [55,56]. Therefore, factor 4 represents natural sources.

In the PMF-1 model, the main elements in factor 1 were Cr and Cu; however, Zn, Pb and Ni have large loads. In PMF-2, the proportion of heavy metal elements in factor 1 was significantly increased. These findings indicate that, due to calculation of PMF, natural sources account for a large proportion, so that factor 1 is the natural source. In factor 2 PMF-1, Cu and Ni account for a large proportion, representing industrial pollution sources; PMF-2 subdivides industrial pollution sources in factor 2 and factor 3. Factor 2 represents atmospheric deposition pollution sources, and factor 3 represents irrigation water pollution sources. Previous studies on the heavy metal input flux of farmland soil reported that the input percentage of Cr from atmospheric deposition was highest and that this was followed by irrigation. The percentage of Ni entering farmland through irrigation was the highest [57]. In PMF-1, the main elements in factor 3 were Cd, Cu, Zn and Pb, in which Cd, Cu and Zn were significantly correlated with SOM content and Pb was the main indicator of traffic emission. Factor 3 was a mixed pollution source of organic fertilizer input and traffic emissions. The main element of factor 4 was Cd, mainly from chemical fertilizer. In PMF-2, the main elements in factor 4 were Cd and Zn, which were the comprehensive sources of organic fertilizer and chemical fertilizer.

### 3.5. Source Contribution Analysis

**Figure 4:**
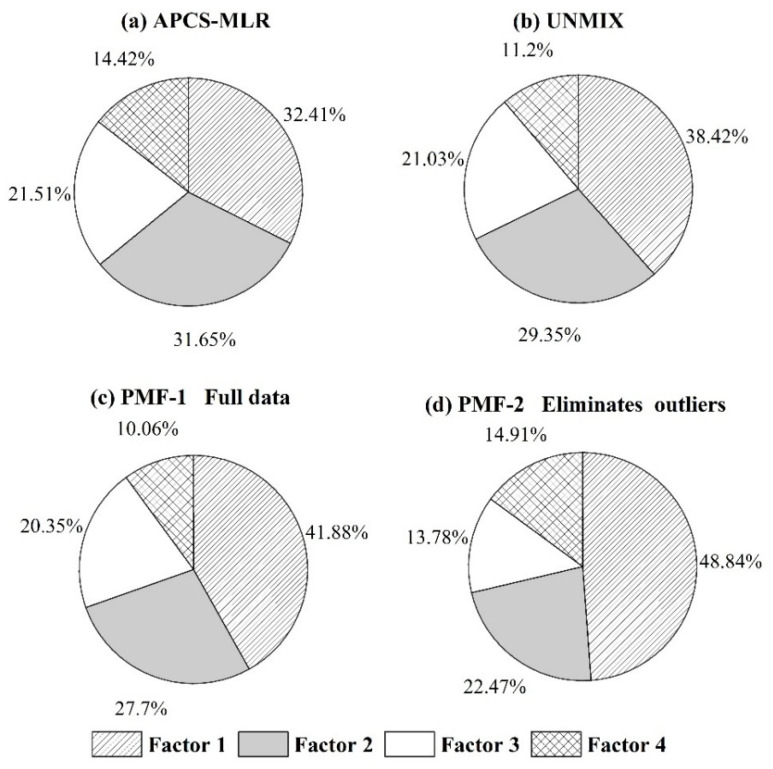
Factor contribution analysis of APCS-MLR, UNMIX and PMF models.

The contribution of each pollution source as calculated by the three models, APCS-MLR, PMF and UNMIX, is demonstrated in Figure 4. The proportions of industrial, agricultural, traffic emission and natural sources in the APCS-MLR model were 32.41%, 31.65%, 21.51% and 14.42%, respectively. The proportions of the above four sources were 38.42%. 29.35%, 21.03% and 11.2% in the UNMIX model. The contributions of natural sources, industrial sources, mixed sources of organic fertilizer and traffic emissions, and chemical fertilizer sources were 41.88%, 27.7%, 20.35% and 10.06%, respectively, in PMF-1. The contribution rates of natural sources, atmospheric deposition, irrigation and fertilizer sources were 48.84%, 22.47%, 13.78% and 14.91%, respectively, in PMF-2.

According to contribution rate of the three models, the results of the APCS-MLR and UNMIX models are consistent. The composition and contribution of pollution sources were highly similar, indicating that the results of source apportionment are reliable. Natural sources accounted for the largest proportion in the PMF model. Except for Cr, other elements in factor 1 have a high loading. The correlation analysis indicated that Cr was significantly correlated with Cu (*p* < 0.05) and correlated with Ni (*p* < 0.1). These findings indicate that Cr, Cu and Ni have common sources. The Zn and Pb in factor 1 were from other pollution sources. It was observed that the fitting effect of Zn and Pb is the worst in the PMF model analysis process (Table 2), which may be due to the wide sources of Zn and Pb in the study area and its high content in the soil. Several areas were interfered with by a variety of pollution sources with a high intensity and long pollution time because the PMF model was unable to accurately identify their pollution sources. The proportion of natural sources in the PMF model increased significantly after the elimination of outliers, suggesting that the PMF model is very sensitive to outliers. The contribution of corresponding pollution sources will be underestimated after the exclusion of outliers. The PMF model can minimize the Q value of the objective function if outliers are retained. The model itself will give the priority to the fitting of outliers. As a result, the factor contribution tends to outliers and accurate source analysis data cannot be obtained. Therefore, the influence of outliers on the analytical results of the PMF model should be carefully considered in the selection of a receptor model.

### 3.6. Evaluation of Potential Ecological Risk Index

The potential ecological risk index has evaluated the pollution of heavy metals in the soil of the study area (Table 5). These analytical results show that the average risk index of heavy metals in the soil of the study area was in the order of Cd (160.01) > Pb (31.94) > Ni (10.69) > Cu (8.60) > Zn (3.85) > Cr (2.56). The mean value of Cd indicated a strong ecological risk degree, and Cd showed a very strong risk degree in a few sampling points. The comprehensive potential ecological risk index ranged from 92.37 to 489.68, with an average value of 217.65. There was a large difference in the risk level, and the overall risk level was medium. The contribution of Cd to the comprehensive potential ecological risk was 73.52%, contributing the main source of the potential ecological risk of heavy metals in the study area.

## 4. Conclusions

The pollution of heavy metals (Pb, Zn and Cd) in farmland soil is more serious due to rapid development of industry and agriculture. Cu, Zn, Pb, Cd and Ni were enriched in the topsoil, whereas Cr was mainly affected by the soil parent material. The potential ecological risk index revealed that Cd was the main element causing the risk of heavy metal pollution in the studied region. The sources of heavy metals in soil were analyzed with the APCS-MLR, UNMIX and PMF models. In the PMF and APCS-MLR models, the industrial sources, agricultural sources, traffic emission sources and natural sources accounted for 32.41% (38.42%), 31.65% (29.35%), 21.51% (21.03%) and 14.42% (11.2%), respectively. These two models were relatively consistent, which could better explain the source and contribution of heavy metal pollution in soil and were more appropriate for this kind of study. However, the PMF model could be easily affected by outliers and the fitting effect of Zn and Pb was not ideal, leading to its high proportion in natural sources, which reduces the credibility of the source apportionment results. The APCS-MLR and UNMIX models were more appropriate for this study. According to the potential ecological risk index, Cd has the greatest ecological risk. Therefore, in the process of soil pollution control, priority should be given to control the harm of Cd to the local ecological environment. The receptor model should be carefully selected in the source apportionment of soil heavy metals. The comprehensive application of multiple models can facilitate the improvement of the reliability of source apportionment results.

## Figures and Tables

**Table 1:** Descriptive statistical analysis of soil heavy metals and soil properties (mg·kg^−1^).

Parameter/ Element	Min	Max	Mean	SD ^a^	CV ^b^(%)	Background of Zhejiang ^c^	Screening Value ^d^	Standard Exceeding Rate (%)
pH	3.94	5.37	4.74	0.29	6.21	——	——	——
SOM ^e^	16.76	66.06	41.65	9.52	22.85	——	——	——
Cu	10.80	57.00	30.26	8.39	27.72	17.6	50.00	3.96
Zn	141.40	532.20	271.50	71.71	26.41	70.6	200.00	86.14
Pb	59.50	361.60	151.41	58.30	38.50	23.7	80.00	97.03
Cd	0.11	0.95	0.37	0.15	40.17	0.07	0.30	70.30
Cr	17.30	159.00	47.81	10.05	21.03	52.9	250.00	0.00
Ni	6.99	71.60	39.07	15.25	39.03	24.60	60.00	0.99

Notes: ^a^ SD stands for standard deviation. ^b^ CV stands for the coefficient of variation, which is the ratio of standard deviation to mean. ^c^ Soil heavy metal background value of Zhejiang province [34]. ^d^ Soil environmental quality risk control standard for soil contamination of agricultural land in China (GB15618-2018). ^e^ SOM means soil organic matter (g·kg^−1^).

**Table 2:** The percentage composition of soil texture (clay, silt and sand) at different depths.

Point	P1			P2			P3			P4		
Depth	Clay	Silt	Sand	Clay	Silt	Sand	Clay	Silt	Sand	Clay	Silt	Sand
0–20 cm	8.5	55.6	35.9	6.2	52.8	41	8.2	46.7	45.1	11.3	46.8	41.9
20–40 cm	10.2	52.4	37.4	8.5	50.4	41.1	5.3	43.1	51.6	13.6	54.2	32.2
40–60 cm	32.5	43.7	23.8	27.6	41.5	30.9	15.6	35.4	49.3	28.2	45.7	26.1
60–80 cm	35.3	45.2	19.5	32.4	43.2	24.4	24.8	38.4	36.8	22.4	38.4	39.2

**Table 3:** Fitting degree r^2^ values of the three receptor models.

Element	APCS-MLR	UNMIX	PMF-1 Full Data	PMF-2 Eliminated Outliers
Cu	0.785	0.880	0.984	0.994
Zn	0.820	0.876	0.402	0.448
Pb	0.924	0.948	0.119	0.213
Cd	0.895	0.914	1.000	1.000
Cr	0.983	0.999	0.403	0.610
Ni	0.906	0.917	0.998	0.995

**Table 4:** Correlation analysis of soil heavy metal content and properties in the study area.

Parameter/Element	pH	SOM	Cu	Zn	Pb	Cd	Cr	Ni
pH	1.000							
SOM	−0.228 *	1.000						
Cu	−0.020	0.430 **	1.000					
Zn	−0.225 *	0.437 **	0.422 **	1.000				
Pb	−0.030	0.130	0.346 **	0.405 **	1.000			
Cd	−0.190	0.487 **	0.170	0.546 **	0.060	1.000		
Cr	0.130	0.090	0.308 **	−0.080	0.010	−0.236 *	1.000	
Ni	0.020	0.395 **	0.567 **	0.277 **	0.090	0.266 **	0.204 *	1.000

* Correlation is significant at the 0.05 level (two-tailed). ** Correlation is significant at the 0.01 level (two-tailed).

**Table 5:** Potential ecological risk index of heavy metals in soil.

Statistics	$E_{r}^{i}$						RI
	Cu	Zn	Pb	Cd	Cr	Ni	
Min	3.07	2.00	12.55	47.14	0.65	2.57	92.37
Max	16.19	7.54	76.29	407.14	9.79	26.32	489.68
Mean	8.60	3.85	31.94	160.01	2.56	10.69	217.65
SD	2.37	1.01	12.24	63.96	1.24	3.38	67.89

## Data Availability

The data presented in this study are available on request from the corresponding author.
